# Nomogram-Based New Recurrence Predicting System in Early-Stage Papillary Thyroid Cancer

**DOI:** 10.1155/2019/1029092

**Published:** 2019-09-05

**Authors:** Yongfeng Ding, Zhuochao Mao, Jiaying Ruan, Xingyun Su, Linrong Li, Thomas J. Fahey, Weibin Wang, Lisong Teng

**Affiliations:** ^1^Cancer Center, The First Affiliated Hospital, Zhejiang University School of Medicine, Hangzhou, China; ^2^Key Laboratory of Precision Diagnosis and Treatment for Hepatobiliary and Pancreatic Tumor of Zhejiang Province, Hangzhou, China; ^3^Department of Orthopedics, The Second Affiliated Hospital, Zhejiang University School of Medicine, Hangzhou, China; ^4^Department of Surgery, New York Presbyterian Hospital-Weill Cornell Medical College, New York, NY, USA

## Abstract

**Background and Objectives:**

The clinicopathological risk factors to predict recurrence of papillary thyroid cancer (PTC) patients remain controversial.

**Methods:**

PTC patients treated with thyroidectomy between January 1997 and December 2011 at the First Affiliated Hospital of Zhejiang University (Zhejiang cohort) were included. Multivariate Cox regression analysis was conducted to identify independent recurrence predictors. Then, the nomogram model for predicting probability of recurrence was built.

**Results:**

According to Zhejiang cohort (*N* = 1,697), we found that the 10-year event-free survival (EFS) rates of PTC patients with early-stage (TNM stages I, II, and III) were not well discriminated (91.6%, 89.0%, and 90.7%; *P*=0.768). The multivariate Cox model identified age, bilaterality, tumor size, and nodal status as independent risk factors for tumor recurrence in PTC patients with TNM stages I–III. We then developed a nomogram with the C-index 0.70 (95% CI, 0.64 to 0.76), which was significantly higher (*P* < 0.0001) than the AJCC staging system (0.52). In the validation group, the C-index remained at a similar level.

**Conclusions:**

In this study, we build up a new recurrence predicting system and establish a nomogram for early-stage PTC patients. This prognostic model may better predict individualized outcomes and conduct personalized treatments.

## 1. Introduction

Thyroid cancer is one of the most common human endocrine tumors. In China, South Korea, and other Asian countries, the incidence of thyroid cancer even increased by 3–6 times in recent decades [1–3]. Among all the thyroid cancer, papillary thyroid cancer (PTC) is the most common pathological type, accounting for about 80–85% patients of total [4, 5]. The majority of PTC patients are indolent and usually have a favorable prognosis. However, a subgroup of PTC patients (about 5%) will develop aggressive growth, metastatic spread and loss of response to conventional therapy [6].

The American Joint Committee on Cancer (AJCC) is the most widely used staging system for thyroid cancer to predict prognosis. Meanwhile, this system is mainly focused on the survival rather than the recurrence, and it therefore is not sufficient to predict recurrence especially for those PTC patients of early stages. The American Thyroid Association (ATA) has put forward a revised recurrence risk stratification system for differentiated thyroid cancer (DTC, including PTC patients) in 2015: the modified 2009 ATA Risk Stratification System (M-2009-RSS) [7], in which they re-divided patients with DTC into high-, median- and low-risk groups [8, 9]. So far, since PTC's unique extended survival period, a number of different staging or prognostic scoring systems were developed, but the controversy remains, particularly on those low-intermediate risk PTC patients.

A nomogram is a visually predictive tool that provides the probability of specific outcomes, such as overall survival and cancer recurrence, for individual patients [10]. Currently, nomograms have been developed in the majority of cancer types, including thyroid cancers [11, 12].

In this study, we found that the EFS of PTC patients with TNM stages I, II, and III was not well discriminated. By using univariate and multivariate Cox regression analysis, we established a new risk stratification system for this specific group of PTCs. A new nomogram was ultimately built to visually predict the probability of recurrence in PTC patients with TNM stages I–III.

## 2. Materials and Methods

### 2.1. Patients and Study Design

A retrospective study was conducted on a primary cohort of patients who underwent total/near-total thyroidectomy for papillary thyroid cancer between January 1997 and December 2011 at the First Affiliated Hospital, Zhejiang University School of Medicine (Hangzhou, China). Patients who had previous radiation exposure, had a family history of PTC, or underwent previous thyroid surgery were excluded. In addition, patients whose follow-up data were not available were excluded. Meanwhile, by reviewing the medical records and pathology reports, these clinicopathologic characteristics of patients were included as follows: age at diagnosis, sex (male and female), bilaterality (yes and no), tumor size (maximum tumor diameter), extrathyroidal extension (including minimal extrathyroidal extension) (yes and no), and nodal status (N0/Nx, N1a, and N1b are defined according to AJCC 7^th^ edition). It should be noted that information related to radioactive iodine (RAI) treatment was not included in the study. This study was approved by the Institutional Review Board of the First Affiliated Hospital, Zhejiang University School of Medicine. Informed consent was obtained before surgery. As we mentioned, all patients were followed postoperatively with measurements of serum thyroglobulin and thyroglobulin antibody, neck ultrasound, and iodine-131 whole-body scans to monitor for disease recurrence and survival [13].

### 2.2. Cox Regression Analysis

Univariate and multivariate Cox regression analyses were conducted to select a subset of independent prognosis predictors for the disease-free survival of PTC. In addition, prognostic index (PI) was calculated using parameters generated by multivariate Cox regression with stepwise selection method.

### 2.3. Construction and Validation of the Nomogram

To construct the effective clinical nomogram, we randomly divided our patients into a modeling cohort and a validation cohort. First, a nomogram was performed using the training cohort based on the independent prognostic factors identified in multivariate Cox regression analysis. The performance of the nomogram was assessed by discrimination (concordance index, C-index) and calibration (comparing nomogram-predicted versus observed Kaplan–Meier estimates of survival probability) [10]. Internal validation of the nomogram was performed using the modeling cohort. The validation cohort was utilized for external validation.

### 2.4. Statistical Analysis

Statistical analysis was conducted using SAS 9.4 (SAS Institute, Inc., Cary, NC, USA). Categorical variables were compared using the *χ*^2^ test or Fisher's test. The optimal cutoff value was determined using X-tile software [14]. Survival curves were depicted using the Kaplan–Meier method and compared by using the log-rank test. Nomogram analysis was performed using the package of rms [15] in R version 3.3.0 (http://www.r-project.org/). For all of the analyses, *P* < 0.05 in a two-tailed test was considered to be statistically significant.

## 3. Results

### 3.1. Deficiency in TNM Staging System

**Figure 1:**
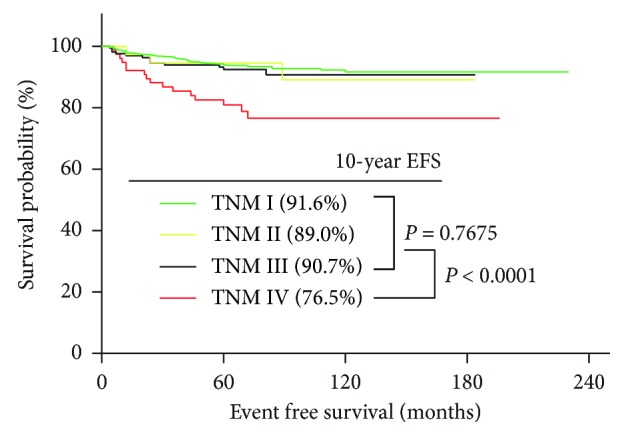
Kaplan–Meier survival curves for event-free survival (EFS) of PTC patients according to TNM stage in Zhejiang cohort. TNM staging in the figure was AJCC 7th edition based.

1697 patients that met the inclusion criteria were included, and the median follow-up time was 67 months (range of 4 to 230 months).  Figure 1 shows that the 10-year EFS rates for TNM I, II, and III were 91.6%, 89.0%, and 90.7%, respectively (*P*=0.768), which were significantly higher than that for PTC patients with TNM IV (76.5%, *P* < 0.0001). These results indicated that TNM staging system was not efficient enough to distinguish the EFS rates for PTC patients with TNM stages I–III.

### 3.2. Clinicopathologic Characteristics of PTC Patients with TNM Stages I–III

1621 PTC patients with TNM stages I–III from Zhejiang Cohort were included for our further analysis. The median follow-up time was 67 months. The demographics and tumor characteristics of patients are summarized in Table 1. The cohort included 384 (23.7%) men and 1237 (76.3%) women with a mean age of 44.1 ± 11.8 years. Bilateral tumors were seen in 303 (18.7%) patients. Patients with the maximum tumor diameter ≤10 mm (papillary thyroid microcarcinoma, PTMC) and >20 mm accounted for 60.9% and 11.1% of the proportion, respectively. The patients with TNM stages I, II, and III were 1420 (87.6%), 37 (2.3%), and 164 (10.1%), respectively.

### 3.3. Cutoff Age for Recurrence Prediction

**Figure 2:**
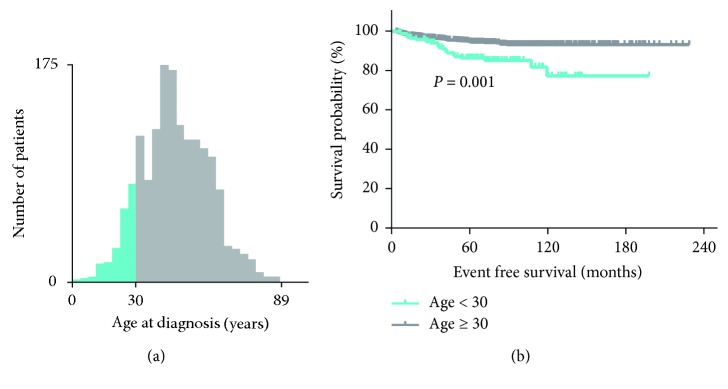
Analysis of age at diagnosis by X-tile software. (a) X-tile determined cutpoint of age and divided patients into high- and low-risk groups. (b) Kaplan–Meier survival curves for EFS according to age stratifications.

To select the best cutoff value of age to predict disease recurrence in PTC patients, the X-tile software was used. The age of 30 was identified as the best cutoff (*P* adjust = 0.001, Figure 2(a)).  Figure 2(b) illustrates the survival curves of EFS for younger than 30 group (age at diagnosis <30) and elder than 30 group (age at diagnosis ≥30) (*P*=0.001; HR = 2.68; 95% CI = 1.72–4.20). Patients who were younger than 30 had a much higher recurrent rate. Thus, we picked age of 30 as cutoff value in our later analysis.

To further assess whether the patients younger than 30 had higher recurrent rate due to lymph node recurrences, we divided the recurrent events into two groups: lymph node recurrence and non-lymph node recurrence (including in situ relapse, contralateral lobe relapse, and distant metastasis). As shown in the supplementary [Supplementary-material supplementary-material-1], the rate of lymph node recurrence in younger PTC group (age <30 y) (8.9%, 15/169) is significantly higher than that in group with age ≥30 y (3.5%, 51/1452, *P* < 0.01). Similarly, the younger group also has higher incidence rate of non-lymph node recurrence (6.0%, 10/169) when comparing with group with age ≥30 y (2.2%, 32/1452, *P* < 0.01). These findings indicate that the elevated recurrence rate of younger patients was possibly due to all-cause recurrence rather than only lymph node recurrence.

### 3.4. Univariable and Multivariable Cox Regression Analyses

**Figure 3:**
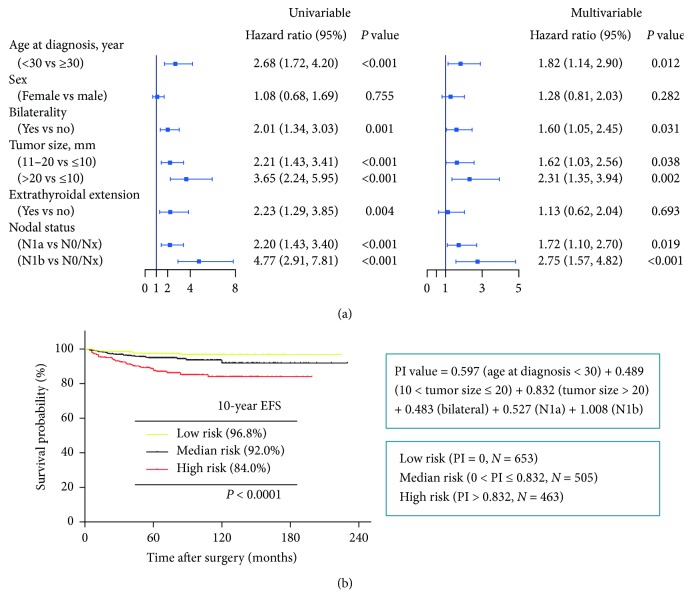
Cox regression analysis and risk stratification for papillary thyroid cancer. (a) Univariate and multivariate cox regression analysis of EFS for papillary thyroid cancer. (b) Kaplan–Meier survival curves for disease-free survival according to risk stratification. The PI value computational formula was based on multivariate Cox regression analysis.

In the univariable Cox regression analysis, we found age at diagnosis, bilaterality, tumor size, extrathyroidal extension, and nodal status were related to EFS (*P* < 0.05) (Figure 3(a)). Sex was not found to be significant. While in multivariate Cox regression analysis, four independent prognostic factors for EFS were identified: age at diagnosis, bilaterality, tumor size, and nodal status. However, extrathyroidal extension was not independently correlated to recurrence (*P*=0.693; HR = 1.13; 95% CI = 0.62–2.04).

### 3.5. Prognostic Index (PI) and New Risk Stratification

Based on the parameters that we generated by multivariate Cox regression, PI values were calculated as follows: PI value = 0.597 (age at diagnosis <30) + 0.489 (10 < tumor size ≤20) + 0.832 (20 < tumor size) + 0.483 (bilateral) + 0.527 (N1a) + 1.008 (N1b). Therefore, we applied the PI to divide PTC patients evenly into high-, median- and low-risk groups. The 10-year EFS rates for the high-, median-, and low-risk groups were well discriminated (84.0% vs 92.0% vs 96.8%, *P* < 0.0001, Figure 3(b)).

### 3.6. Construction of the Nomogram and Validation

**Figure 4:**
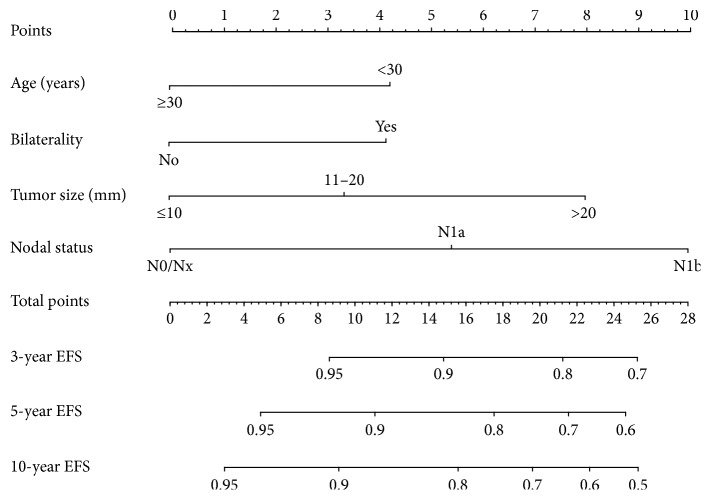
The nomogram for predicting event-free survival (EFS) of papillary thyroid cancer (the nomogram was used as follows: possible values of each variable from an individual patient are located on each variable axis, and a line is drawn upward to determine the points got for each variable on the point axis. The sum of these points is located on the total point axis. Then, a line is drawn downward to the survival axes to determine the likelihood of 3-, 5-, or 10-year EFS. A specific example is shown in Supplementary [Supplementary-material supplementary-material-1]).

We constructed a nomogram to develop a new predictive tool for calculating probability of EFS at the individual level based on the factors identified as significant independent variables in the multivariate Cox regression analysis reported above. We randomly divided 1621 patients with TNM stages I–III into the modeling group (*n* = 1215) and the validation group (*n* = 406) at the rate of 3 : 1. There were no significant differences between the modeling group and validation group in all 6 clinicopathologic characteristics (*P* > 0.05, Supplementary [Supplementary-material supplementary-material-1]). By integrating all significant independent factors for EFS, we constructed the nomogram based on the modeling data set (Figure 4). The discrimination (C-index) of our nomogram for EFS prediction was 0.70 (95% CI, 0.64 to 0.76), which was significantly higher (*P* < 0.0001) than the AJCC staging system (0.52). The calibration plot for the probability of EFS at 3, 5, or 10-year showed an optimal agreement between the prediction by our nomogram and actual observation (Supplementary Figures [Supplementary-material supplementary-material-1]–[Supplementary-material supplementary-material-1]). In addition, in the external validation using the validation group, the C-index was 0.65 (95% CI, 0.55 to 0.75), and a calibration curve showed good agreement between prediction and observation in the probability of 5-year survival (Supplementary [Supplementary-material supplementary-material-1]).

## 4. Discussion

In this study, we found that the widely used TNM staging system is not efficient enough to predict tumor recurrence in PTC patients with TNM stages I, II and III. By calculating prognostic indexes based on a multivariate Cox model, we further constructed a new risk stratification system, in which the 10-year EFS rates for the low-, median-, and high-risk groups were well discriminated. Finally, we developed a new nomogram with high C-index to better estimate individualized prognosis for early-stage PTC patients.

There exists a long-lasting debate on whether and how the age influences the prognosis of thyroid cancer. Orosco et al. analyzed 85,740 patients in the SEER database and found no single year between 25–55 can be chosen to yield a high hazard ratio for survival, which suggests none of them should be served as a unique cutoff [16]. Since the majority of PTCs have good prognosis with high survival rate of 10 and even 20 years, the factors that affect recurrence should be better delineated. Our data identified the age of 30 as the optimal cutoff for recurrence and was further confirmed as an independent risk factor by the multivariable Cox regression analysis. Similarly, Cho et al found the recurrence rates of PTC were higher in patients under 35, among which 15.7% of recurrences were under the age less than 25 years [17]. Here, we found that PTC patients of young age had higher potential risk of recurrence. For this group of patients, we suggest increasing the intensity and frequency of follow-up.

Bilateral PTC has been traditionally discovered after total thyroidectomy with the incidence varying from 20% to 60% according to the literature [18]. However, few data specifically examining the prognostic implications of bilaterality in PTC have been reported. Pellegriti et al. first evaluated the association between bilaterality and recurrence risk in 299 PTCs [19]. Subsequently, we detailed this relationship in 891 PTC patients [20] and further confirmed in 2,211 patients in the later follow-up study [13]. We found that bilateral PTC is common, occurring in 19.9% of our cohort, and that bilaterality is associated with poorer prognosis. Our data presented here are the first to identify bilaterality as an independent risk factor for recurrence in PTC, supporting total thyroidectomy as the preferred surgical approach in patients with bilateral disease. In a recent study of 3282 patients with micro-PTC, Hwangbo et al. also found bilaterality as an independent risk factor for long-term recurrence [21]. The clonality study from our group revealed most bilateral PTC tumors are of similar clonal origin, suggesting this specific disease is a consequence of a single primary with subsequent intrathyroidal metastasis to the contralateral thyroid lobe. This observation provides a molecular rationale as to why the outcome of bilateral PTCs was observed to be poorer in this dataset [22].

The AJCC staging system has been criticized for its inability to accurately predict the prognosis of PTC patients, especially for the low risk majority [23, 24]. While the incidence of PTC has increased dramatically in recent decades, the mortality has remained relatively unchanged, implying that most of the increase is due to low-risk PTCs [2, 5, 25]. Distinguishing the aggressive low-risk tumors from the indolent majority could help to avoid overtreatment of the low-risk PTCs. Various risk stratification systems have been proposed, such as the MACIS (metastasis-age-completeness of resection-invasion-size) prognostic scoring system [26], and the ATA active surveillance system [7]. Nomograms also been applied to predict cancer-specific survival or recurrence of thyroid cancer [11, 27]. In addition, studies have reported that nomograms resulted in more accurate prognostic prediction than the traditional staging systems for patients with colon, stomach, and liver cancer [28–30]. In the current study, we developed a prognostic nomogram for predicting recurrence risk in PTC patients with TNM stages I–III. To our knowledge, this is the first risk stratification system that specifically evaluates recurrence in PTC patients with TNM stages I–III. When compared with conventional TNM staging system, the nomogram showed better predictive accuracy for recurrence, and external validation showed that the model had good stability.

The limitation of our study is that it was a retrospective, single-institution research. Due to the higher proportion of microcarcinoma in our study, the conclusions of this study are limited and need to be strictly verified in groups with relatively low proportion of microcarcinoma, such as Europe and the United States population. And, the TNM data were based on the 7th edition of the AJCC staging system. The newly published 8th edition possesses some changes including the increased age cutoff from 45 to 55 y and the removal of the minimal extrathyroidal extension from the definition of T3 [31]. However, AJCC staging was mainly developed to predict risk for death and the benefits of 8th edition in predicting recurrence which we focused in this study remain unknown [32, 33]. We plan to further compare the difference of EFS between these two staging systems in our later study. Another limitation is the lack of some other important variables that could be informative, such as information on RAI treatment and the genetic alteration status (BRAF, RAS, TERT etc). We plan to validate the new risk stratification system by some cohorts outside and to further combine genetic alterations like BRAF and TERT promoter mutations in that system.

## 5. Conclusion

In conclusion, this study provides initial evidence that the EFS of early-stage PTC patients (TNM stages I, II, and III) cannot be well discriminated. By using univariate and multivariate Cox regression analysis, we established a new risk stratification system for this specific group of PTCs.

## Figures and Tables

**Table 1:** Demographics and clinicopathologic characteristics of patients with papillary thyroid cancer of TNM stages I–III (*N*  =  1621).

Demographic or characteristic	No. of patients	%
Age at diagnosis, years	44.1 ± 11.8^a^	—
	44 (8, 89)^b^	—
Sex		
Male	384	23.7
Female	1237	76.3
Bilaterality		
Yes	303	18.7
No	1318	81.3
Tumor sizec (mm)		
≤10	988	60.9
11–20	453	28.0
>20	180	11.1
Extrathyroidal extension		
Yes	108	6.7
No	1513	93.3
Nodal status		
N0/Nx	1150	70.9
N1a	360	22.2
N1b	111	6.9
TNM stage		
I	1420	87.6
II	37	2.3
III	164	10.1

^a^Mean ± standard deviation; ^b^median (range); ^c^maximum tumor diameter.

## Data Availability

The data used to support the findings of this study are included within the article.

## Supplementary Materials

Supplementary Materials Supplementary Table 1: comparison of the rate of lymph node recurrence and non-lymph node recurrence in different age groups. Supplementary Table 2: comparison demographics and clinicopathologic characteristics of patients with papillary thyroid carcinoma. Supplementary Figure 1: an example for the nomogram model; a 50-year-old female with bilateral PTC, whose tumor size is between 10 and 20 mm and nodal status is N1b. Her total score in this nomogram is about 17.58, and 3-, 5- and 10-year EFSs are about 86%, 80%, and 76%, specifically. Supplementary Figure 2: the calibration curve for predicting patient EFS survival at (A) 3 years, (B) 5 years, and (C) 10 years in the modeling cohort. *X*-axis, nomogram-predicted probability of EFS; *Y*-axis, actual EFS. Supplementary Figure 3: the calibration curve for predicting patient survival at 5 years in the validation cohort.
