# Supplementary material for: Nomogram-Based New Recurrence Predicting System in Early-Stage Papillary Thyroid Cancer
Source: Int J Endocrinol. 2019 Sep 5;2019:1029092. doi: 10.1155/2019/1029092 (PMC6754965; doi:10.1155/2019/1029092)
Supplement: Supplementary Materials — Supplementary Table 1: comparison of the rate of lymph node recurrence and non-lymph node recurrence in different age groups. Supplementary Table 2: comparison demographics and clinicopathologic characteristics of patients with papillary thyroid carcinoma. Supplementary Figure 1: an example for the nomogram model; a 50-year-old female with bilateral PTC, whose tumor size is between 10 and 20 mm and nodal status is N1b. Her total score in this nomogram is about 17.58, and 3-, 5- and 10-year EFSs are about 86%, 80%, and 76%, specifically. Supplementary Figure 2: the calibration curve for predicting patient EFS survival at (A) 3 years, (B) 5 years, and (C) 10 years in the modeling cohort. X-axis, nomogram-predicted probability of EFS; Y-axis, actual EFS. Supplementary Figure 3: the calibration curve for predicting patient survival at 5 years in the validation cohort. [file 1029092.f1.docx]

| Supplementary Table 1. Comparison of the rate of lymph node recurrence and non-lymph node recurrence in different age groups. | | | |
| --- | --- | --- | --- |
|  | ＜30y | ≥30y | P-Value |
| Lymph node recurrence / all patients | 15/169 | 51/1452 | 0.00079 |
| Non-lymph node recurrence / all patients | 10/169 | 32/1452 | 0.00403 |

| Supplementary Table 2. Comparison demographics and clinicopathologic characteristics of patients with papillary thyroid carcinoma. | | | | | | |
| --- | --- | --- | --- | --- | --- | --- |
|  |  |  |  |  |  |  |
| Demographic or Characteristic | Primary Cohort (N = 1215) | |  | Validation Cohort (N = 406) | | *P* value^a^ |
|  | No. of Patients | % |  | No. of Patients | % |  |
| **Age at diagnosis, year** |  |  |  |  |  |  |
| <30 | 158 | 13.00 |  | 43 | 10.59 | 0.202 |
| ≥30 | 1057 | 87.00 |  | 363 | 89.41 |  |
| **Sex** |  |  |  |  |  |  |
| Male | 280 | 23.05 |  | 104 | 25.62 | 0.292 |
| Female | 935 | 76.95 |  | 302 | 74.38 |  |
| **Bilaterality** |  |  |  |  |  |  |
| Yes | 231 | 19.01 |  | 72 | 17.73 | 0.567 |
| No | 984 | 80.99 |  | 334 | 82.27 |  |
| **Tumor size，mm** |  |  |  |  |  |  |
| ≤10 | 733 | 60.33 |  | 255 | 62.81 | 0.480 |
| 11-20 | 349 | 28.72 |  | 104 | 25.62 |  |
| >20 | 133 | 10.95 |  | 47 | 11.58 |  |
| **Extrathyroidal extension** |  |  |  |  |  |  |
| Yes | 79 | 6.50 |  | 29 | 7.14 | 0.654 |
| No | 1136 | 93.50 |  | 377 | 92.86 |  |
| **Nodal status** |  |  |  |  |  |  |
| N0/Nx | 870 | 71.60 |  | 280 | 68.97 | 0.390 |
| N1a | 260 | 21.40 |  | 100 | 24.63 |  |
| N1b | 85 | 7.00 |  | 26 | 6.40 |  |

Tumor size, maximum tumor diameter; a, χ2 test.


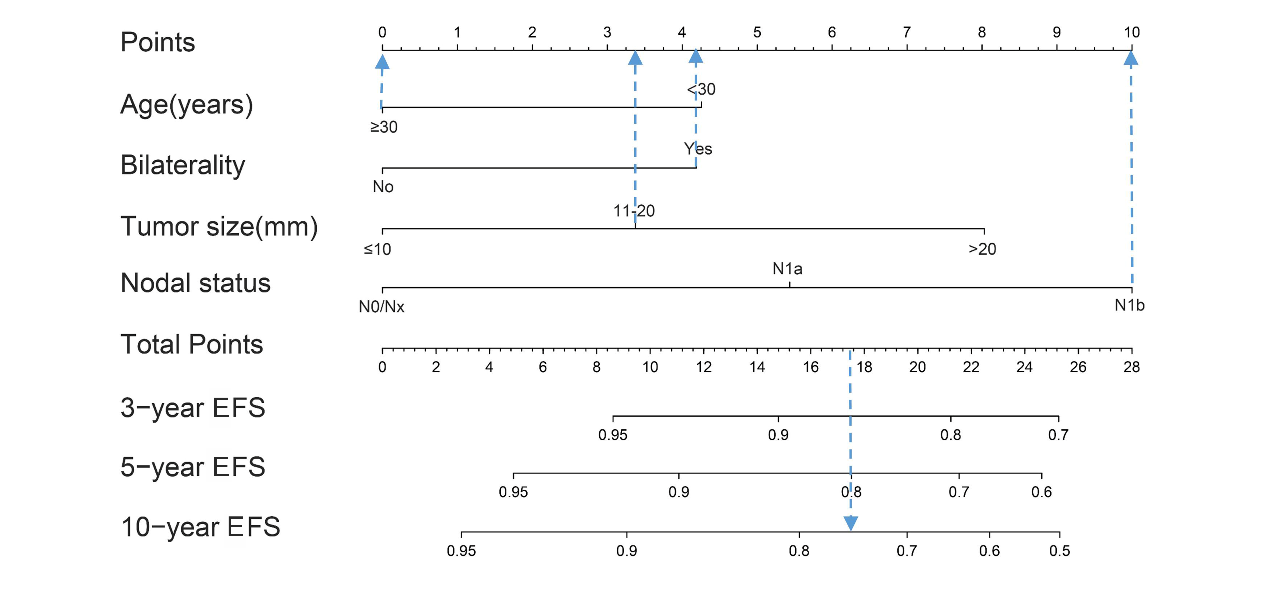


Supplementary Figure 1. An example for the nomogram model. A 50 years old female with bilateral PTC, whose tumor size is between 10 and 20 mm and nodal status is N1b. Her total score in this nomogram is about 17.58 and 3-, 5- and 10-year EFSs are about 86%, 80% and 76%, specifically.


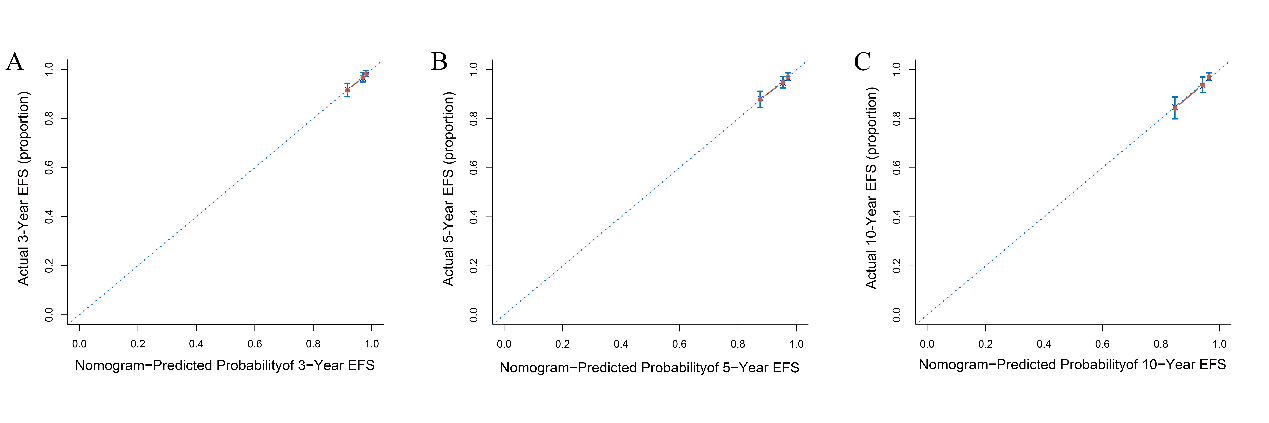


Supplementary Figure 2. The calibration curve for predicting patient EFS survival at (A) 3 years, (B) 5 years and (C) 10 years in the modeling cohort. X-axis, nomogram-predicted probability of EFS; Y-axis, Actual EFS.


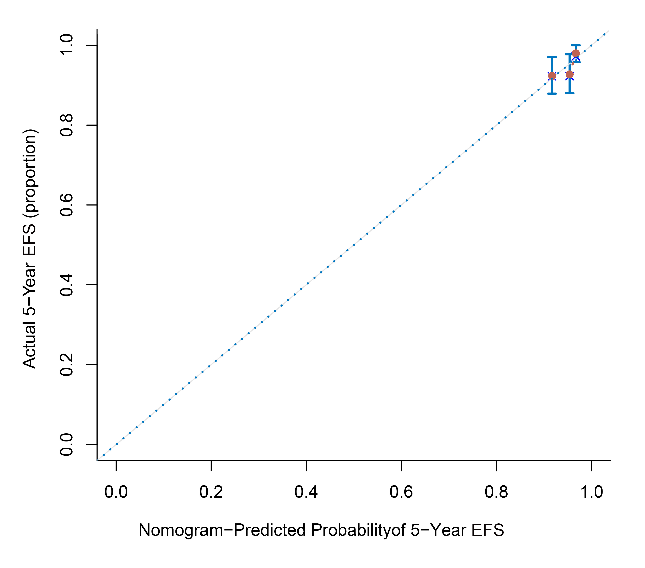


Supplementary Figure 3. The calibration curve for predicting patient survival at 5 years in the validation cohort.
